# Surviving at Any Cost: Guilt Expression Following Extreme Ethical Conflicts in a Virtual Setting

**DOI:** 10.1371/journal.pone.0101711

**Published:** 2014-07-09

**Authors:** Cécile Cristofari, Matthieu J. Guitton

**Affiliations:** 1 Institut Universitaire en Santé Mentale de Québec, Quebec City, Quebec, Canada; 2 Faculty of Medicine, Laval University, Quebec City, Quebec, Canada; University of Vienna, Austria

## Abstract

Studying human behavior in response to large-scale catastrophic events, particularly how moral challenges would be undertaken under extreme conditions, is an important preoccupation for contemporary scientists and decision leaders. However, researching this issue was hindered by the lack of readily available models. Immersive virtual worlds could represent a solution, by providing ways to test human behavior in controlled life-threatening situations. Using a massively multi-player zombie apocalypse setting, we analysed spontaneously reported feelings of guilt following ethically questionable actions related to survival. The occurrence and magnitude of guilt depended on the nature of the consequences of the action. Furthermore, feelings of guilt predicted long-lasting changes in behavior, displayed as compensatory actions. Finally, actions inflicting immediate harm to others appeared mostly prompted by panic and were more commonly regretted. Thus, extreme conditions trigger a reduction of the impact of ethical norms in decision making, although awareness of ethicality is retained to a surprising extent.

## Introduction

The study of moral dilemmas has always faced an unresolved challenge: ethical concerns forbid to use anything else than hypothetical scenarios, where one might doubt the sincerity of participant responses. Unfortunately, the question of how people would react if they had to solve a moral dilemma in an actual emergency situation is far from idle, nor is it only relevant to philosophy or moral cognition. Indeed, while many countries in the world enjoy a comparatively safe daily life, the possibility of major unplanned crises – be they caused by a natural disaster, pandemics, or a war – represents a threat that modern societies cannot ignore [1], [2], [3], [4]. In such a situation, people might have to make drastic choices that could jeopardize their lives or others’ on a regular basis [5].

In practice, however, the study of moral dilemmas is limited by the nature of the devices used to test them [6]. Most studies use relatively standardized protocols in which participants are given a description of a scenario involving a life-or-death situation that can be solved in different ways, usually according to either utilitarian or ethical principles [7], [8], [9], [10], [11]. While such protocols have the advantage of being easily settled and controlled in a laboratory, they dramatically lack an ecological component [11], [12], [13]: answers given in a safe environment in which no choice will have real consequences are likely to be an imperfect reflection of how people would react to a similar situation in real life.

Immersive virtual spaces could represent a solution to this challenge, by providing alternative models to test human behavior in controlled life-threatening situations [4], [14], [15], [16], [17], [18]. Indeed, people have been demonstrated to display stronger emotional reactions in response to virtual reality rather than text [19]. Moreover, reactions to virtual persons have been found to be similar to reactions to people in real life [20], a fact that reinforces the potential usefulness of virtual settings to study moral dilemmas. Previous studies in moral psychology have used virtual reality to test pre-defined scenarios, thus creating a more immersive environment to increase the engagement of the participants [19], [21]. However, a further step could be taken by dispensing with pre-defined scenarios altogether, in order to improve the ecological aspect of the experimental setting. Of particular interest would be immersive virtual worlds featuring the aftermath of catastrophic events – for instance a zombie outbreak – which would closely mimic the fundamental characteristics of any large-scale existential risk event [4], [22], [23], and where life-threatening situations forcing users to make drastic choices would spontaneously emerge. In this context, the recently developed massively multiplayer online survival game DayZ could represent an interesting model [4]. The game mechanics of DayZ allow a great degree of freedom in avatar behavior; at the same time, however, the survivalist logic of the setting forcibly constrains the range of actions that can safely be undertaken. Observing the in-game behavior of user-controlled survivors could represent an extremely powerful empirical way to assess possible discrepancies between moral values and acts, or shifts in moral values under experienced – in contrast to purely theoretical – life-threatening conditions.

While accessing the instantaneous behavior of hundred of thousands of survivors in the game on a continuous basis would be infeasible in practice, users themselves provide a solution to this problem, by reporting many of their actions in the game forum. Being entirely spontaneous, and neither prompted nor required, those self-reports can be taken as a genuine expression of their authors’ experiences and reactions to them. It could be hypothesized that if participants experienced guilt following ethically questionable actions, they would self-report it when telling the anecdote. Accessing such behavioral sequences through self-reports in forum posts could thus offer a very strong insight on moral dynamics. Using the forum could allow to test more specific points, in particular whether guilt will be more often reported following actions with immediate consequences than actions with delayed consequences, if the presence of guilt would predict the occurrence of actions undertaken specifically as a way to compensate for the harm done, and how ethically problematic actions are justified.

The aim of the present study is not to draw generalized conclusions about the behavior of DayZ users from a restricted sample, but rather, to use self-reports from forum users to investigate human behavior in response to moral dilemmas in extreme situations. Based on the analysis of more than a hundred different self-reported situations, we investigated the dynamics of moral control of actions and guilt perception under extreme conditions. The occurrence and magnitude of self-reported guilt depended on the nature of the action’s consequences. Our results also reveal that extreme conditions may trigger a reduction of the impact of ethical norms in decision making, although awareness of ethicality is retained to a surprising extent.

## Methods

### 1. Model

The free open world survival horror game DayZ (originally a mod designed for the tactical shooter game ARMA 2) has over one and a half million registered users (www.dayzmod.com). This immersive post-apocalyptic setting consists in an imaginary country plagued by zombies, where survivors (embodied by the users) have to wrestle their surroundings to find food, weapons or medical supplies, while their lives are under the permanent threat of zombies or other hostile survivors. In contrast to most other games which allow a dead character to be instantly resurrected (or “respawned”) at a little distance, with their equipment and experience level intact, death in DayZ results in much more dramatic losses. Characters start again from scratch in a random place without previously gained equipment and food. Thus, death affects the character’s situation far more than is customary in most online games, forcing users to be much more cautious with their characters. As a result, user behaviors tend to be more realistic [4]: they can choose to either attack other characters, or team up with them in order to increase their own chances at survival. Betrayal is common, selfish behaviors can be observed on a regular basis, and so can acts of altruism and loyalty. An online forum (www.dayzmod.com/forum) allows users to share experiences and discuss anecdotes and viewpoints. It is common for users to ask for or receive comments on whether their actions were justified or ethical, leading to a number of conversations on ethics or behavioral norms in general.

### 2. Data collection

All materials were obtained from the public access official forum of the DayZ mod (www.dayzmod.com/forum). Threads from the “General Discussion” and “Bandit Campfire” sections, last updated from 1^st^ January 2013 to September 26^th^ 2013, were systematically searched for posts describing an action that the author of the post undertook in-game in response to a situation presenting a possible ethical choice, and that displayed a level of awareness of the possibility of an ethical judgement. All threads were searched, unless obviously out of topic (e.g., threads dealing with in-game bugs, “out of character” threads started by players looking for teammates). The posts selected had to tell a specific anecdote rather than describe generalities. There was no specific criterion for length or complexity. Posts were selected when authors acknowledged the possibility of guilt (either recognising feeling guilty or explicitly stating the opposite). In order to avoid bias, posts where the author stated that they did not feel guilty were not automatically discarded, but were instead coded as such. Posts that did not explicitly mention the possibility of guilt, but were part of a thread dedicated to anecdotes about ethically problematic in-game actions (e.g. “*Things you were not proud of*”), were included, as the awareness of an ethical question was considered implicit.

Posts that referred to a general feeling but did not tell a specific anecdote were not included. In order to select posts that displayed comparable levels of immersion, posts that dismissed ethical concerns by explicitly stating such an issue had no place in a game setting, posts that referred to killing as “fun” in a gaming context, as well as posts that were obviously sarcastic were not taken into account in the present selection. All coding was performed by an observer blind to the hypotheses.

### 3. Author and post characterization

For each post author, the following characteristics were recorded: total number of posts, date of registration on the forum, frequency of posting (number of posts per day), self-reported gender and geographical origin. Data were compared to the average activity of the members of the forum, assessed as the frequency of posting of the first 500 users selected by alphabetical order. The size (number of words) of each post as well as the presence or absence of humorous markers (verbal such as jokes, or non-verbal such as emoticons) were recorded.

### 4. Self-reported moral judgement

For each post, individual actions involving an ethical challenge were identified and analysed independently. Individual actions were characterised according to the following criteria. First, the consequences of the considered action were recorded depending on whether the action had directly caused another character’s death (“immediate”), or resulted in a situation where death was hardly avoidable in the long term, for instance by robbing a character of weapons or supplies, or by wounding them severely (“delayed”). In those cases, although they did not witness the final consequences of their action, the perpetrators could not ignore what would be the fate of their victims. The intensity of the reported feelings of guilt was recorded as well. Three levels were identified: “Guilty”, “Somewhat guilty” and “Not guilty”. Action descriptions followed by a clear expression of definite guilt were categorized as “Guilty”, action descriptions followed by ambiguous, mitigated or possibly euphemistic expressions of guilt (“*I feel kinda bad*”, “*I’m not especially proud*”) were marked “Somewhat guilty”, and action descriptions that clearly stated that there were no feelings of guilt were characterized as “Not guilty”. Fine-tuning of reaction assessment was made more difficult by the language used in posts (e.g. does “I felt bad” refer to guilt or shame?). Therefore, we did not attempt to differentiate between guilt and shame.

When indicated, the reason of guilt was labelled as “in character” if the guilt originated in feelings related to the played character, either directly after the action had taken place, or following the acquisition of *a posteriori* information, for instance when contextual knowledge was revealed and demonstrated that the killing was not necessary. Conversely, guilty feelings were labelled as “out of character” when the author of the action did not feel particularly bad about the act of killing another character, but did feel bad about degrading the gaming experience of another human player. When mentioned in the post, the justification of the action was recorded as emotional (for example a reaction to fear), utilitarian (for instance involving a desire to appropriate the other character’s possessions to facilitate one’s survival), or other (judgements, such as stating that the character “deserved” to be harmed for a certain reason, or composite explanations involving emotional, utilitarian and judgemental elements). The last recorded item was the presence or absence of compensatory actions taken by the author of the post after the initial ethically problematic action (for example, apologizing to the other user, or helping them after they came back to the game with a different avatar).

### 5. Identification of user-defined norms of behavior

A second selection of posts was realised in order to identify norms of behavior related to ethics. 50 threads were selected from the “General Discussion”, “Bandit Campfire” and “Survivor HQ” sections, between April 23^rd^, 2013 and October 14^th^, 2013, corresponding to the 50 most recent threads relevant to our question. Threads were selected when they explicitly asked a question about ethics (e.g. “*Is it wrong to…?*”), or when they featured an anecdote followed by judgements from the author or other people (e.g. “*You did the right thing*”), from which implicit or explicit norms could be inferred. Threads having less than 10 posts were discarded. Both explicit and implicit norms of behavior were subsequently recorded, along with the number of times they appeared. When the same user reiterated an opinion on a norm of behavior in the same thread, that second expression of their opinion was not taken into account. However, when the same users displayed the same opinion in several threads, it was recorded every time. The total number of times a single norm was mentioned across all 50 threads was recorded. To account for the possibility that the same user might mention the same norm in many different threads, therefore skewing the statistics, we counted the total number of users who took part in all the threads from our sample, the number of threads each user had taken part in, and the average number of threads per user.

### 6. Statistical analysis

Due to the nature of the data gathered, a non-parametric approach was privileged. Therefore, Mann-Whitney U tests were used to compare groups in pairs. Given the limited number of occurrence in some of the sub-categories considered, z-tests were favored over Chi-square tests to compare proportions. When applicable, all data are presented as mean ± SEM.

## Results

### 1. Characteristics of the main corpus

A total number of 126 posts by 119 authors telling anecdotes and referring to ethical concerns were identified, representing a total 149 single ethically problematic actions. In addition, 5 purely benevolent actions were identified during the sampling (2 of them belonging to posts describing ethically problematic actions). Due to the survivalist nature of the game, which imposed a definite logic to the types of actions that could be taken by the avatars, ethically problematic actions always had to do with either directly killing a user-embodied character, or putting them in mortal danger. Anecdotes were thus all comparable in terms of their nature: finding oneself in perceived life-threatening danger, having to make a choice, occasionally taking impulsive action. They could therefore all be analysed using the same parameters, and compared on the same bases.

On average, the authors of the considered posts had registered 315.4±11.8 days ago and posted 384±112.9 times on the forum. In contrast, while the first 500 active users of the forum by alphabetical order had registered 388.5±5.6 days ago, they had published only 29.8±4.2 posts. Thus, the users from our sample were significantly more active on the forum than the average user (Mann-Whitney U test, U = 10451.50, *p*<0.001). The majority of the authors identified themselves as male (55%, while 45% users did not disclose their gender). 57.14% did not disclose their geographical localisation, 6.72% indicated a place from the game, 28.57% indicated an English-speaking country (USA, UK, Australia, Canada, Ireland) and the remaining 7.56% stated coming from various countries, mostly European. Posts were on average 211.91±16.38 words long (3.69±0.38 paragraphs per post).

### 2. Actions with immediate vs. delayed consequences

123 actions out of 149 had immediate detrimental consequences for the victims, usually resulting in their death (“Immediate”, 82.55%). In contrast, 26 actions out of 149 (e.g. robbing a character of their supplies and letting them go defenceless, or severely harming a character and leaving them without cure) had delayed consequences (“Delayed”, 17.45%), which would likely be lethal due to the extremely hostile nature of the environment.

### 3. Self-reported feelings of guilt

**Figure 1:**
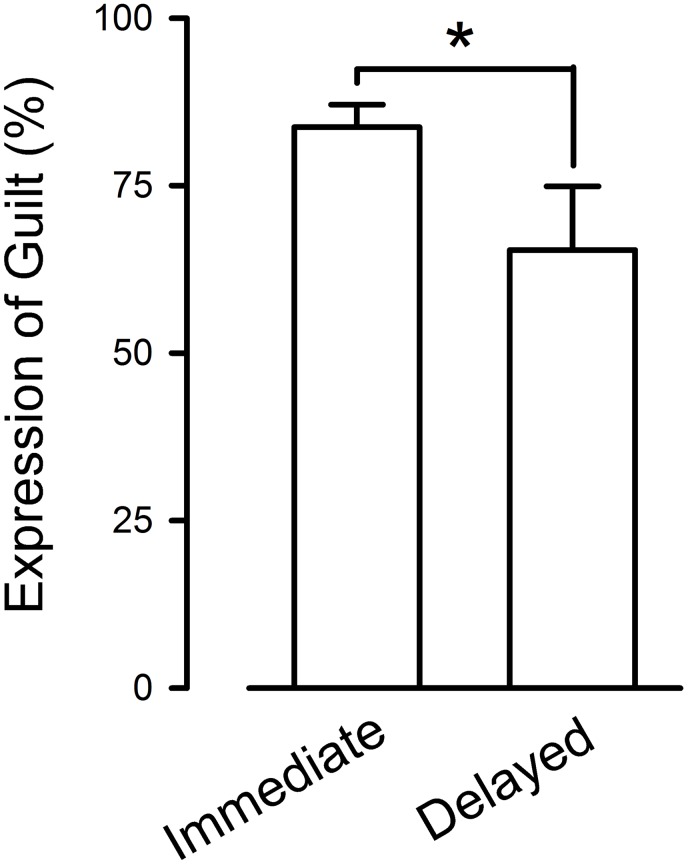
Occurrence of self-reported guilt following ethically problematic situations depending on the consequences of the action. *indicates p<0.05.

A total of 120 situations presented a self-report of feelings of guilt following the action taken (80.54%). The remaining 29 were coded “Not guilty”. Guilt was never expressed at killing zombies. In the Immediate group, 103 actions were followed by self-reports of guilt (73 Guilty and 30 Somewhat guilty), and 20 were not (Not guilty). In the Delayed group, 17 actions were followed by self-reports of guilt (13 Guilty and 4 Somewhat guilty), and 9 were coded as Not guilty. Actions against a character in the game that had immediate detrimental consequences were significantly more guilt-inducing than actions having delayed consequences (83.74±3.34 and 65.38±9.51 for actions with immediate and delayed consequences, respectively; Mann-Whitney U test, U = 1305.50; *p*<0.05, **Figure 1**).

**Figure 2:**
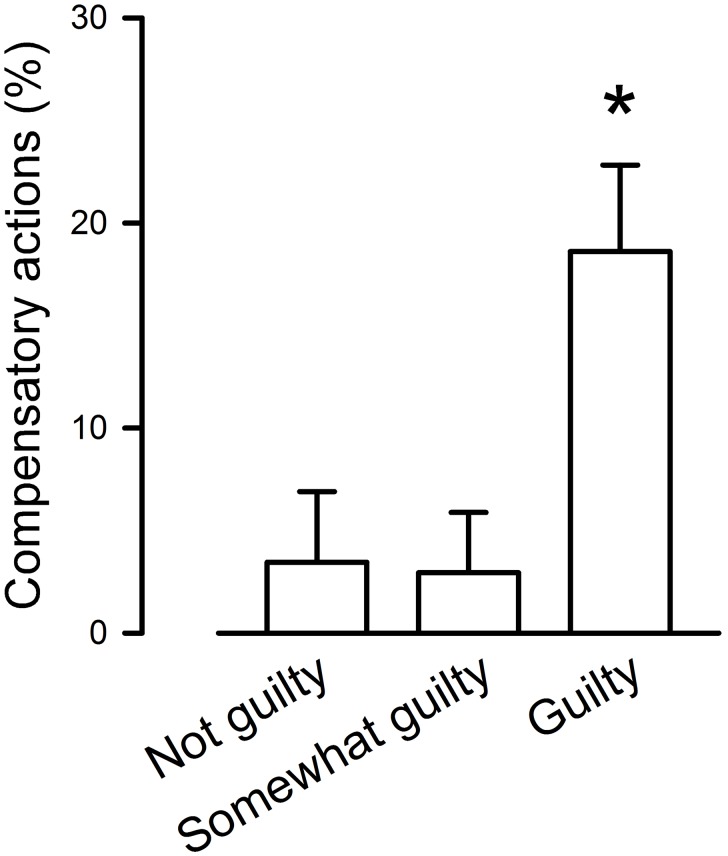
Occurrence of compensatory actions following ethically problematic situations depending on the intensity of the self-reported guilt. *indicates p<0.05.

Actions taken in order to compensate for the harm done (“compensatory actions”) following the ethically questionable decisions were self-reported in 18 (12.08%) of the ethical situations evidenced in the posts. While the number of self-reported compensatory actions was not different when comparing Guilty+Somewhat guilty (frequency: 0.14±0.03) to Not guilty (frequency: 0.03±0.03; Mann-Whitney U test, U = 1553.50, *p* = 0.11), significantly more compensatory actions took place when only taking into account the Guilty group (excluding the Somewhat guilty group), and comparing them to situations the Not guilty group (frequency: 0.19±0.04; Mann-Whitney U test, U = 1058.00, *p*<0.05, **Figure 2**).

### 4. Presence of justifications

The vast majority of ethically questionable situations were accompanied by some form of justification (118 out of 149, 79.19% vs. 20.81% of the situations without justification). The two main categories of justifications were the “emotional” justifications (e.g., “*I shot because I panicked*”, 34 actions, 22.82% of the total corpus, 28.81% of the actions presenting a justification) and the “utilitarian” justifications (e.g., “*I needed his possessions, so I shot him*”, 69 actions, 46.31% of the total corpus, 58.47% of the actions presenting a justification). Only very few actions were justified by other reasons (either judgements or composite explanations, 15 actions, 10.07% of the total corpus, 12.71% of the actions presenting a justification).

**Figure 3:**
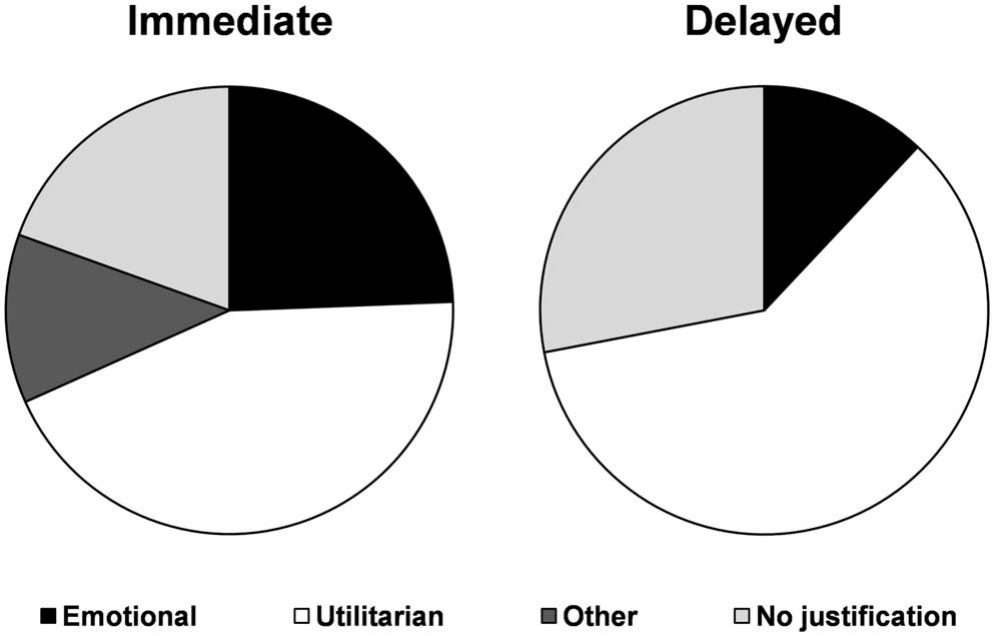
Nature of the justifications of the ethically problematic actions depending on their consequences (immediate, left vs. delayed, right, repartitions significantly different *p*<0.05).

Justifications were not found more commonly following actions inducing guilt than actions not inducing guilt (*p* = 0.63). No statistical relationship appeared either when breaking down the justifications into four categories (88.24% with guilt and 11.76% without guilt, *p* = 0.2 for emotional justification; 79.71% with guilt and 20.29% without guilt, *p* = 0.82 for utilitarian justifications; 73.33% with guilt and 26.67% without guilt, *p* = 0.46 for other justifications; and 23.33% with guilt and 76.67% without guilt, *p* = 0.55 for no justification). However, the study of the overall distribution of the explanations as a function of the immediate vs. delayed characteristics of the consequences showed significant differences (z-test, z = 2.19, *p*<0.05, **Figure 3**).

When mentioning the reasons why culpability arose, users described either reasons related to the character (“in character”, users felt guilty for having killed or harmed another character, 55.03%), or to the player himself, as the human behind the screen (“out of character”, users felt guilty for making the game less enjoyable for another user, 22.82%). “In character” reasons were more frequently given than “out of character” reasons (significantly, z-test, z = 5.58, *p*<0.001).

### 5. Analysis of norms of behavior

The total number of posts in 50 threads selected as having a topic related to ethical concerns was 2036. The number of relevant posts (excluding posts that did not contribute to the discussion of behavior norms, for example posts containing only emoticons) was 639 (31.39% of the total posts). 586 users were recorded across all 50 conversations. 366 of them took part in only one conversation, and the average number of conversations per user was 2±0.08. A total of 60 norms of behavior were found. Those norms could be positive (e.g. “*It is acceptable to kill in self-defense*”) or negative (e.g. “*It is not acceptable to commit betrayal*”). Most of the time, however, they could not be paired as binary opposites, since they frequently expressed nuances in the permissibility of a given behavior (revealing distinctions between what was considered permissible, permissible but frowned upon, unacceptable or obligatory behavior).

The vast majority of the norms were “in character” (65% norms, detailing how survivors should or should not behave, from an ethical or utilitarian point of view) compared to “out of character” (35% norms related for instance to fair play or to considerations of what behavior should be displayed by avatars based on the user’s views of human nature, a difference significant at *p*<0.05, assessed by z-test, z = 3.10).

## Discussion

### 1. Validity and limitations of the experimental model

A major and common issue with experimental settings aiming at exploring human moral cognition is that safe environments in which no choice will have real consequences are likely to lead to an imperfect reflection of how people would react in an actual life-or-death situation [9], [12], [13], [24]. Moreover, the difference between egocentric and allocentric points of view, with on the one hand the possibility for the subjects to suffer from the consequences of their choices, and on the other hand a simple judgement on the situation without self implication, is of major importance when discussing the notion of the moral vs. utilitarian value of an action [25], [26], [27]. Even a first-person story is not likely to elicit the same level of involvement – much less the same level of guilt – as a situation one would directly experience. Using an existing game and spontaneous productions of strongly involved users presents multiple ecological advantages. First, a variety of behaviors can be expressed, with no direction from the researchers, thus eliminating observer-expectation bias. Second, focusing on users significantly more active than average entirely removes the bias due to varying levels of familiarity with the interface, as seasoned users can all be assumed to be proficient with the game mechanics. Finally, while participants confronted with a hypothetical scenario in a laboratory setting may feel little investment with it, and their answers may therefore be influenced by a number of other factors (e.g. a desire to give a socially acceptable, or what could be perceived as a “right”, answer), people using a virtual game setting of their own volition are much more likely to be immersed, and therefore, to give authentic responses.

Thus, one of the main advantages of virtual environments is their immersive potential, i.e. the possibility for users to truly “embody” the avatar they control, leading to an experience comparable to a lived experience [14], [28], [29]. It has been shown that emotional responses elicited by a virtual environment could, with caution, be generalized to the real world [28], [30]. Reactions to a moral dilemma experienced in an immersive virtual environment could therefore help reduce the inaccuracies that are due to the purely theoretical nature of story-based moral dilemmas.

However, the extent to which this immersion process takes place in the present virtual environment is an important concern regarding the validity of zombie apocalypse settings as a model to study moral cognition and human behavior in response to large-scale crises. Would people react to the experiences of an avatar in a virtual space in the same way as they would react if faced with the same dilemma in real life? Indeed, zombie narratives are not realistic in the strictest sense, as zombies are not part of the real world. However, several lines of argument still support a positive answer. It has previously been demonstrated that lack of strict realism in a virtual world is not a problem in itself, and that a virtual world can be immersive even when it is blatantly unrealistic [29], [31]. Furthermore, the focus of DayZ is not actually the zombies, but the survival mechanisms in a hostile environment, as well as player-versus-player interactions (which are, in themselves, more strictly realistic than interactions with zombies, as they involve human avatars in a situation of realistic conflict). While choices made in the game may not mirror real-life situations, and do not have consequences in the real world, users are likely to react to the situations they encounter in the virtual world as if they were real. Supporting this is the fact that users in DayZ can possibly embody their characters in the virtual world for weeks, leading to a very strong feeling of attachment, reinforced by the vulnerability of the virtual character. Because the anecdotes analysed were self-reported and entirely spontaneous, they can be understood as a genuine expression of the users’ reactions. Furthermore, the details of those reports themselves paint a picture that is consistent with the hypothesis that the feelings of guilt were authentic, and the very occurrence of compensatory actions suggested that the feeling of guilt was real.

Another important argument comes from the prevalence of “in character” over “out of character” feelings in the self-reported anecdotes. Whether we investigated feelings of guilt or spontaneously expressed norms of ethical behavior, “in character” elements were significantly more frequently named than “out of character” elements, a fact that cannot be attributed to a small number of users expressing their opinion in many different posts due to the low average number of conversations from our sample users took part in. This reinforces the notion that the characters and their predicament are acknowledged as worthy of far more consideration than mere fictional artefacts would, and that, therefore, the treatment they receive could be close to the way an actual human might be treated. This general prevalence of “in character” over “out of character” strongly suggests that our model is valid. Interestingly, this was also the insight of some users, as some “out of character” norms took as a fundamental assumption that avatars in the game ought to mimic normal human behavior. Hence, the sample we accessed might not have been representative of the general population of the users of the game, but was unbiased regarding the specific objectives of the present study – which was to identify human behavior in response to crisis situations. Due to their high level of immersion, it is highly probable that the behavior displayed by the users of our sample was isomorphic to the behavior they would have displayed in a similar real-life situation.

In the present study, the behavior of DayZ users was assessed via self-reports. Like any methodology, this way of collecting behavioral material presents a few limits. While self-reports spontaneously given on a forum can be interpreted as a genuine expression of the users’ reactions, they may be influenced by the user’s personal interpretation of their own reactions at the time. Furthermore, the forum gave us no means to assess how much time had elapsed between the incident experienced by the user, and the report on the forum; variations in time elapsed might affect a posteriori perception of the event. In the present case, this bias is likely to have been compensated to a large extent by the size of our sample. Nonetheless, this method of using spontaneously generated self-reports presented a major ecological advantage compared to self-reports which could have been generated in laboratory conditions.

### 2. Expressions of guilt in extreme conditions

While several emotions can be related to moral judgement, guilt has been consistently reported as an important emotion in the development of moral insights [32], or a motivator of actions in a moral dilemma situation [33]. Actions with immediate consequences caused more guilt than actions with delayed consequences. This is consistent with the general insight of moral psychology, which has demonstrated that people find the idea of directly harming another person less acceptable than the idea of undertaking an action that will indirectly cause their death, even if the outcomes are the same [6], [7], [34]. The greater importance of emotional justifications for “Immediate” actions, undertaken without preparation as a reaction to a sudden crisis, also confirms the validity of moral psychology findings obtained in laboratory conditions, which suggested that emotional reactions play an extremely important role in ethical decision-making – possibly greater than moral reasoning [35], a finding confirmed by the importance of emotional over utilitarian justifications for the “Immediate” actions in our study – especially when the dilemma involve directly harming someone [7], [10], [36]. However, while previous studies emphasised the role of emotions in moral judgement [37], [38], [39], our findings show evidence of the importance of emotions in the moral judgment-related decision-making process itself, and of the fact that emotions may be a particularly prominent factor when choosing a course of action that could result in direct harm to someone.

Self-reported guilt appeared to follow distinct patterns. The immediacy of the consequences of the action predicted more self-reports of guilt. While it would be legitimate to question whether those feelings were genuine (instead of being, for instance, a convention in the narration of anecdotes on the forum), the fact that users reported modifying their own behavior as a consequence of those feelings, and that stronger reported feelings of guilt did increase the likelihood of compensatory actions, strongly suggested that those feelings were real. What is more, compensatory actions were significantly linked to unambiguous expressions of guilt only, this statistical significance disappearing when taking into accounts self-reports of mitigated guilt. Therefore, the intensity of guilt varied with the perceived gravity of the action, and immediate consequences were associated with greater gravity in the minds of the users.

The study of the justifications revealed that actions with immediate consequences were more likely than actions with delayed consequences to have an emotional justification, while actions with delayed consequences were more likely to be triggered by utilitarian concerns. The higher occurrence of expressions of guilt following actions with immediate consequences suggests that those actions had more psychological impact on those who performed them [36]. It is interesting to notice that even in the most drastic conditions, survivors appeared reluctant to kill another human for purely utilitarian reasons, in line with an evolutionary logic of human ethics [24]. In contrast, not directly witnessing the death of another human – even if virtual – seemed to abolish this natural inhibition, at least partially. In spite of the extreme conditions emulated by the environment of DayZ, what was perceived as acceptable behavior (regardless of the actual actions undertaken) appeared to be largely similar to real-world norms.

However, we observed a clear dichotomy between actual actions (what the users did when facing the online situation) and their moral judgements (how they reflected on their own actions afterward). Indeed, the extremely hostile environment might trigger a disruption of inhibitions, leading to a discrepancy between moral judgement and actual actions. In that situation, the quick alterations of human reactions may mask the capacity of moral norms to remain relatively conserved: the construction of a set of norms of behavior tending towards moral norms in the specific environment of DayZ appears to differ relatively little from the established real-world norms. While those results do no preclude the possibility of a shift in moral cognition following a long-term exposure to an extremely hostile environment, they suggest that important inhibitions would have to be overcome for such a shift to happen. The question whether these inhibitions are purely cultural or bear biological bases remains unanswered.

While our chosen experimental model enabled us to get valid results, further studies might be needed to overcome its limitations. DayZ focuses on survival, without focusing on the creation of social bonds, or reconstruction of any kind. However, our present results already suggest that extreme in-world conditions appear to have a behavioral impact, as users regularly rob, harm or kill. Ethical concerns nonetheless remain surprisingly important, and much of the immediate harm done by users is prompted by panic and immediately regretted, rather than though-out. While our study focused on expressions of guilt, such a model could be further used to develop tools for predicting human behaviors in times of crisis, to explore whether ethical norms would remain the same or undergo a progressive shift under long-term exposure to drastic conditions.
